# Serpentine and Vermiform Are Produced Autonomously to Fulfill Their Function in *Drosophila* Wings

**DOI:** 10.3390/insects14050406

**Published:** 2023-04-23

**Authors:** Xubo Zhang, Yanan Ji, Bernard Moussian, Shumin Yang, Jianzhen Zhang, Tingting Zhang, Min Zhang

**Affiliations:** 1 Shanxi Key Laboratory of Nucleic Acid Biopesticides, Research Institute of Applied Biology, Shanxi University, Taiyuan 030006, China; zhangxubo@sxu.edu.cn (X.Z.);; 2 INRAE, CNRS, Institut Sophia Agrobiotech, Sophia Antipolis, Université Côte d′Azur, 06108 Nice, France

**Keywords:** *serpentine*, *vermiform*, wing, fat body, cuticle permeability

## Abstract

**Simple Summary:**

In *Drosophila melanogaster*, *DmCDA1* (*Serpentine*, *serp*) and *DmCDA2* (*Vermiform*, *verm*) are responsible for wing cuticle barrier establishment. In the embryo, Serp is produced in the fat body and is transported to tracheal cells to participate in tracheal development. To answer the question as to whether these CDAs in the wing tissue were produced locally or derived from the fat body, we inhibited *serp* and *verm* specifically in the fat body or the wing primordia. We revealed that Serp and Verm were produced autonomously in the wing, and repression of these two enzymes caused wing defects locally. They fulfill their function in the wing independently of their expression in fat body. This study advances the understanding of the role of chitin deacetylase in insect wing development.

**Abstract:**

Group I chitin deacetylases (CDAs), CDA1 and CDA2, play an essential role in cuticle formation and molting in the process of insect wing development. A recent report showed that trachea are able to take up a secreted CDA1 (*serpentine*, *serp*) produced in the fat body to support normal tracheal development in the fruit fly *Drosophila melanogaster*. However, whether CDAs in wing tissue were produced locally or derived from the fat body remains an open question. To address this question, we applied tissue-specific RNAi against DmCDA1 (*serpentine*, *serp*) and DmCDA2 (*vermiform*, *verm*) in the fat body or the wing and analyzed the resulting phenotypes. We found that repression of *serp* and *verm* in the fat body had no effect on wing morphogenesis. RT-qPCR showed that RNAi against *serp* or *verm* in the fat body autonomously reduced their expression levels of *serp* or *verm* in the fat body but had no non-autonomous effect on the expression in wings. Furthermore, we showed that inhibition of *serp* or *verm* in the developing wing caused wing morphology and permeability deficiency. Taken together, the production of Serp and Verm in the wing was autonomous and independent of the fat body.

## 1. Introduction

Wings of insects play essential roles in flight and courtship. Most of insect wings are composed of a bi-layered membrane structure [1]. A main component of the wing cuticle is the polysaccharide chitin, which interacts with chitin-binding proteins to form a quasi-crystalline structure that is implicated in the shape and function of various tissues including the wing. Chitin deacetylation is crucial for the proper function of the cuticle, which requires the activity of chitin deacetylases (CDAs; EC 3.5.1.41). CDAs are chitin-modifying enzymes that deacetylate chitin to form chitosan [2,3,4,5,6,7]. CDAs are located at the assembly zone of epidermal cuticle suggesting that chitin deacetylation may be coupled with chitin synthesis in this zone [8]. CDA coding genes have been found in fungi, arthropods and nematodes [9,10,11]. Insect CDAs are classified into five groups according to the sequence similarity and domain diversity [7,12]. Chitin deacetylases are glycoproteins and possess signal peptide; they are predicted to be secreted proteins [13]. Group I CDAs, including CDA1 and CDA2, have been reported to play an indispensable role in chitinous tissue differentiation [6,12,13,14,15,16,17,18,19,20].

In the red flour beetle *Tribolium castaneum*, suppression of *TcCDA1* or *TcCDA2* caused molting failure during larval–larval, larval–pupal and pupal–adult stages. The cuticle lost its normal laminae organization in *T*. *castaneum* with reduced *TcCDA1* or *TcCDA2* transcript levels [21]. In *D*. *melanogaster*, *DmCDA1* (*serpentine*, *serp*) and *DmCDA2* (*vermiform*, *verm*) are involved in the formation and extension of trachea during in the embryo [18]. We previously reported that Serp and Verm are responsible for wing cuticle barrier establishment and that Serp is required for chitin deacetylation in the wing cuticle [17].

Dong et al. [22] demonstrated that Serp was secreted by the fat body to regulate the development of the tracheal system in the embryo. In general, the insect fat body serves as an energy reservoir, releasing and delivering components to target tissues to regulate normal biological processes [23]. Here, we asked whether fat body derived Serp and Verm may contribute to wing development. In other words, are these two enzymes produced locally in the wing epithelial cells, or are they provided by the fat body?

To study this problem, we knocked down the transcript levels of *verm* and *serp* using the Gal4/UAS system in developing wings and in the fat body. Knockdown of *serp* or *verm* in wing cells resulted in morphological defects and reduced barrier efficiency as previously reported [17]. Suppression of *serp* or *verm* in the fat body did not cause any obviouswing defects. Furthermore, we showed that repression of these two genes in the wing posterior compartment affected the permeability of the posterior compartment but not of the anterior compartment. Thus, the function of Serp and Verm in the wing tissue is independent of their expression in the fat body. Our findings facilitates deeper understanding of the role of chitin deacetylase in the insect wing development.

## 2. Materials and Methods

### 2.1. Drosophila Stocks

The following stocks were used in this study: *UAS*-*verm*-RNAi (v15464) and *UAS*-*serp*-RNAi (V15466) were purchased from Vienna Drosophila Resource Center (VDRC), and cg-GAL4 (BL#7011) was obtained by Bloomington Drosophila Stock Center (BDSC). The other stocks *tub*-Gal80^ts^, hh-Gal4, nub-Gal4, and UAS-*brinker* were provided by Prof. Jie Shen at China Agricultural University.

### 2.2. RNAi of Serp and Verm

For specific repression of *serp* or *verm* in the fat body, cg-Gal4 stock was crossed with *UAS*-*verm*-RNAi or *UAS*-*serp*-RNAi to produce F1 generations as cg > *verm*-RNAi or cg > *serp*-RNAi, respectively. For local repression of genes in the posterior compartment of the wing, *tub*-Gal80^ts^, hh-Gal4 fly stock was crossed with *UAS*-*verm*-RNAi or *UAS*-*serp*-RNAi fly stocks. The according genotype of F1 were *tub*-Gal80^ts^, hh *> serp*-RNAi or *tub*-Gal80^ts^, hh *> verm*-RNAi. The crossed flies were cultured at 18 °C, and F1 generations were transferred to 30 °C from late second instar larval stage until adult emergence.

### 2.3. Adult Wing Observation

In brief, the adult wings were collected 48 h after eclosion. The wings were emerged in 75% ethanol and washed on a shaker for 5–6 times with 6–8 min for each time for removing debris and dust on their surface. The cleaned wings were placed on a glass slide and embedded in glycerol. They were observed using a Multifocus Imaging System of Fluorescence Microscope (MV PLAPO 1×, Olympus, Tokyo, Japan) equipped with a CCD camera (DFC450 C, Leica, Wetzlar, Germany) for imaging.

### 2.4. Reverse Transcription Quantitative PCR (RT-PCR)

Total RNA from the wing discs of wandering larvae (30 wing discs for each sample) and white pupa (100 wing buds for each sample, 36 h after pupation at 30 °C) were extracted using RNAiso^TM^ Plus (TaKaRa, Maebashi, Japan), respectively. A total of 1 μg of total RNA was used to synthesize the first-strand cDNA using M-MLV Reverse Transcriptase (Promega, Madison, WI, USA) with oligo-(dT) 18 primer (TaKaRa, Maebashi, Japan). cDNA of each group was diluted 40 times for RT-qPCR analysis. The primers for quantification of *serp* and *verm* transcript levels were used as previously reported [17]. The reaction solution includes the contents as follows: 10 μL of SYBR Green qPCR Master Mix (TOYOBO, Osaka, Japan), 4.4 μL of deionized water, 4 μL of diluted template and 0.8 μL of 0.4 μM forward and reverse primers, respectively. The qPCR was performed on an ABI 7300 real time PCR machine (Applied Biosystems, Waltham, MA, USA) with the following program: denaturation at 95 °C for 1 min followed by 40 cycles at 95 °C for 15 s, 60 °C for 31 s. We used the melting curve to determine the gene-specific peak and the signal for primer-dimers for each sample. The 2^−ΔCT^ method was applied to quantify the gene expression level using *rp49* and *rps20* as control. For each sample, three independent biological and two technical repetitions were conducted. Statistical analysis between groups was based on Student’s *t*-test.

### 2.5. Cell Apoptosis Detection in Wing Discs

The 3rd instar larvae were dissected, and their wing discs were fixed in 4% formaldehyde solution for 40 min. The samples were rinsed with PBT for four times and kept in PBT for 1 h on a rocking shaker. Next, samples were incubated with the Caspase-3 primary antibody (1:100) at 4 °C overnight. Then, they were rinsed with PBT for four times and kept in PBT for another 30 min before incubation with the secondary antibody for 1 h. Samples were rinsed with PBT for four times and kept in fresh PBT for another 1 h. Wing discs of nub > *brinker* flies were used as the positive control. Finally, images were collected by an inverted Fluorescence Microscope (EVOS FL, Life Technologies, Carlsbad, CA, USA).

### 2.6. Eosin Y Penetration Assay

Three-day-old flies were collected for the Eosin Y penetration assay [24,25]. The flies were incubated in 1 mL dye solution (0.5% Eosin Y [*w*/*v*] and 0.1% Triton X-100) at 55 °C for 30 min. They were washed thereafter for three times with distilled water. Wings were removed from the body and mounted onto glass slides. Images were taken by a MV PLAPO 1× microscope/Multifocus Imaging System of Fluorescence Microscope.

### 2.7. FB28 Staining

Fluorescent brightener 28 (FB28) is widely used as a chitin-detection dye [26,27]. The wing samples were cut along the A/P compartment boundary, and half of the wings were fixed in 4% formaldehyde solution for 8 h, then rinsed with PBT 4 times and washed with PBT for 2 h on a rocking shaker. Samples were incubated with FB28 (Sigma, Ronkonkoma, NY, USA) (1 mg/mL) for 5 min. The images were taken by an inverted Fluorescence Microscope (EVOS FL, Life Technologies, Carlsbad, CA, USA).

## 3. Results

### 3.1. Knockdown of Serp and Verm in the Fat Body Does Not Cause Wing Deficiency

**Figure 1:**
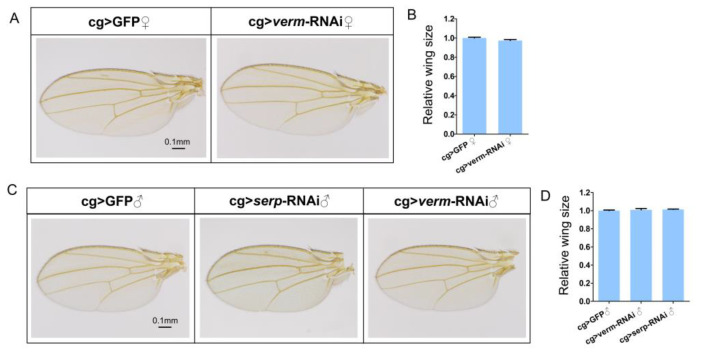
Repression of *serp* or *verm* in fat body using cg-Gal4 driver had no effect on wing development. Adult wings of cg > GFP, cg > *serp*-RNAi and cg > *verm*-RNAi flies. The morphology of the wings of both control and RNAi groups were indistinguishable (**A**,**C**). The sizes of female or male wings did not show significant differences to the control wings (**B**,**D**). The scale bar is 0.1 mm. More than 20 wings were used in every group.

It was previously reported that Serp was secreted from the fat body into the hemolymph and taken up by the tracheal cells where it contributed to tracheal development [22]. To investigate the origin of Serp and Verm, which we have shown to function in wing development [17], we reduced their transcript levels in the fat body by expressing *serp* and *verm* specific dsRNA under the control of cg-Gal4 driver. Similar to cg *>* GFP flies, the wings of cg *> serp*-RNAi and cg > *verm*-RNAi flies did not show any visible phenotype (Figure 1A,C) neither in males nor in females. We next compared the wing size in control and in cg > *serp*-RNAi or cg > *verm*-RNAi flies. The wing size was not significantly different between cg *>* GFP and cg *> serp*-RNAi flies (Figure 1D). Similarly, cg *> verm*-RNAi flies displayed a normal wing size (Figure 1B,D). These results indicate that knockdown of *serp* or *verm* in the fat body does not affect wing morphology and size.

### 3.2. Knockdown of Serp and Verm in the Fat Body by cg-Gal4 Has no Effect on the Expression Level of Serp and Verm in the Wing

**Figure 2:**
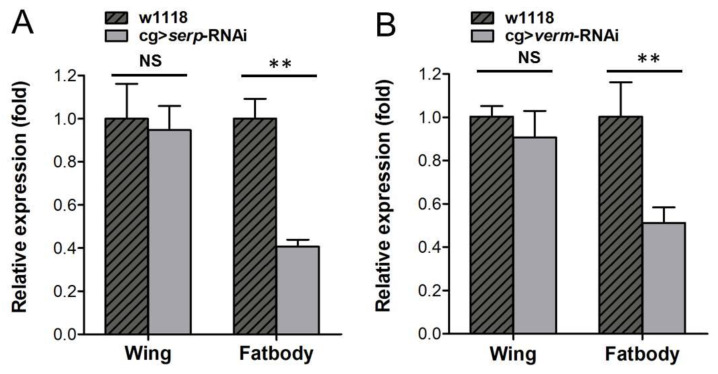
RNAi of *serp* and *verm* in fat body did not affect expression levels in wing: *serp* and *verm* mRNA levels in the fat body and in wings were tested for cg > *serp*-RNAi (**A**) and cg > *verm*-RNAi flies (**B**) The relative levels of *serp* and *verm* in the fat body were decreased significantly but did not have an effect on their expression in wings. Wings were dissected at the second day after pupation, while the fat body was dissected from wandering larvae. RNAi efficiency for *serp* or *verm* was assayed by RT-qPCR. Asterisks indicate significant differences (*p* < 0.01).

To determine the RNAi efficiency of *serp* and *verm* in the fat body, we used RT-qPCR to analyze the transcript levels of *serp* and *verm* both in the fat body and the wing bud. The relative levels of *serp* and *verm* in the fat body were significantly decreased in cg > *serp*-RNAi or cg > *verm*-RNAi flies (Figure 2A,B). Next, we sought to rule out the possibility that fat body-specific RNAi against *serp* and *verm* transcripts also influenced the transcript levels in the wing. We found that both *serp* and *verm* transcript levels in the wing tissue were unchanged in the respective RNAi flies compared to the control (Figure 2A,B). These results suggest that cg > *serp* (*verm*)-RNAi flies suppressed the transcript levels of target genes in the fat body but did not affect them in wings. This result implies that the production of Serp and Verm in the wing is independent of the fat body.

### 3.3. Knockdown of Serp and Verm in Posterior Areas Caused Wing Deficiency In Situ

**Figure 3:**
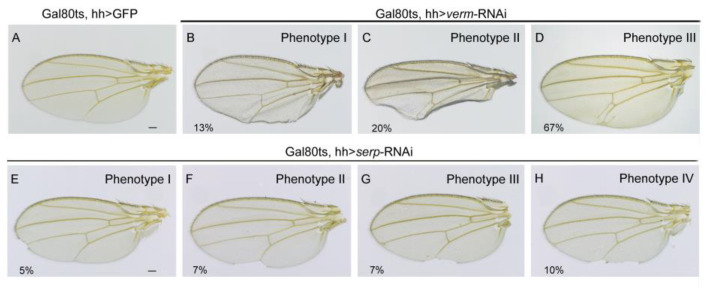
Repression of *serp* or *verm* in the posterior compartment caused deficiency in the posterior but not in the anterior compartment of the wing. (**A**) Wings of Gal80^ts^, hh-Gal4 > GFP were used as control. (**B**–**D**) Wings of Gal80^ts^, hh-Gal4 > *verm*-RNAi flies displayed three types of phenotypes in the posterior compartment. Phenotype I: wings were curly (**B**); Phenotype II: ectopic veins appeared (**C**); Phenotype III: wings were curly and ectopic veins appeared (**D**). (**E**–**H**) *serp*-knockdown flies (Gal80^ts^, hh-Gal4 > *serp*-RNAi) exhibited four types of incised wing phenotypes in the posterior compartment. The scale bar is 0.1mm. At least 35 wings were analyzed for each genotype.

To confirm that the function of Serp and Verm during wing development is tissue-autonomous, we used the wing posterior compartment-specific hh-Gla4 to express the UAS-RNAi transgenes. The wings of Gal80^ts^, hh > *verm*-RNAi flies showed three phenotypes. In particular, 13% of wings were curly at the posterior area (Figure 3B); 67% of wings displayed an ectopic vein at the posterior cross-vein (p-cv) position (Figure 3D); and 20% of wings showed both phenotypes (Figure 3C). However, together, only 29% of the wings of Gal80^ts^, hh > *serp*-RNAi flies showed a phenotype (Figure 3E–H). Bristles were missing, and the wing border was nicked in the posterior regions of wings at different sites. In general, the knockdown of *serp* or *verm* in the posterior area caused wing defects in the posterior compartment, whereas the anterior compartment was intact.

### 3.4. Wing Deformations Caused by Serp or Verm Suppression Were Not Due to Cell Apoptosis of Wing Disc

**Figure 4:**
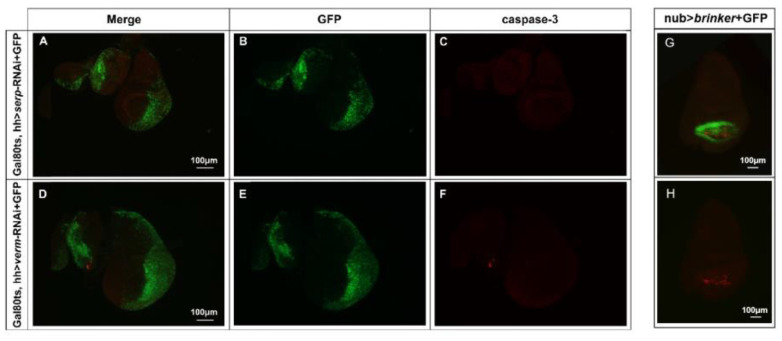
Repression of *serp* and *verm* in the posterior wing region did not induce cell apoptosis in wing disc. Cell apoptosis in the wing discs of the *serp*- and *verm*-knockdown flies was detected by anti-Caspase-3 detection (**A**–**F**). The Caspase-3 signal was not detected in the wing disc of both *serp* and *verm* RNAi flies (**C**,**F**). Wing discs of nub > *brinker* + GFP were used as a positive control for the anti-Caspase-3 staining (**G**,**H**). More than 20 wing discs were used for every genotype. The scale bar is 100 μm.

To detect whether the wing phenotype was due to increased cell death, we examined possible apoptosis events in the wing disc after *serp* and *verm* suppression. An anti-Caspase-3 antibody was used to detect cell apoptosis. The wing disc in *Gal80ts*, hh *> verm*-RNAi or in *Gal80ts*, hh *> serp*-RNAi larvae did not show any Caspase-3 signal, indicating that there is no induced apoptosis caused by *serp* or *verm* suppression (Figure 4A–F). Overexpression of *brinker* in the pouch region of wing discs has been shown to induce cell death. Here, we used nub > *brinker* as a positive control for the caspase-3 staining. Hence, wing deformations caused by the *serp* or *verm* knockdown was not related to increased cell apoptosis in wing discs.

### 3.5. Repressing Serp and Verm in the Posterior Compartment Had no Non-Autonomous Effect on the Barrier Function of the Anterior Compartment

**Figure 5:**
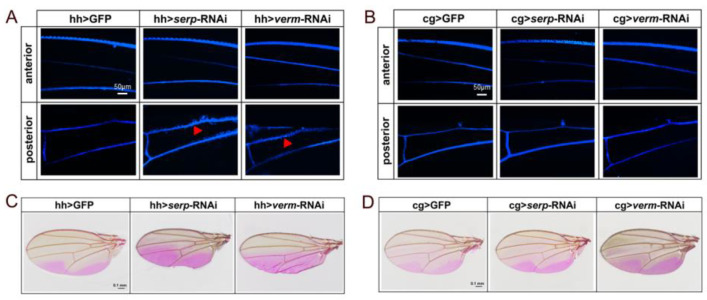
Repression of *serp* and *verm* in the posterior wing compartment induced modification of wing permeability. In control wings, the veins of whole wings are straight, and FB28 did not penetrate the regions between veins. Diffusion of FB28 at the veins was promoted in the posterior area of the wings of Gal80^ts^, hh *> serp*-RNAi and Gal80^ts^, hh *> verm*-RNAi flies, red triangles indicate the lateral FB28 dye diffusion (**A**). In cg > GFP, cg > *serp*-RNAi and cg > *verm*-RNAi flies, FB28 did not diffuse from the veins (**B**); the scale bar is 50 μm (**A**,**B**). (**C**,**D**) Wings took up Eosin Y in penetration assays at 55 °C. (**C**) The areas of Eosin Y penetration expanded to the whole posterior compartment after inhibiting *serp* or *verm* under the control of hh-Gal4 driver. The Eosin Y staining regions of cg > *serp*-RNAi and cg > *verm*-RNAi wings were similar to the control (**D**). The scale bar is 0.1 mm (**C**,**D**), more than 15 wings were used for each group.

We performed FB28 staining to detect wing cuticle barrier efficiency in the wing. The FB28 signal around the L4 and L5 veins of the posterior compartment was diffused in *Gal80^ts^*, hh > *verm*-RNAi and *Gal80^ts^*, hh > *serp*-RNAi flies (Figure 5A). This indicates that the wing structure was disrupted allowing lateral FB28 dye diffusion. By contrast, the wing veins in anterior compartment in *Gal80^ts^*, hh > *verm*-RNAi and *Gal80^ts^*, hh > *serp*-RNAi wings exhibited a sharp boundary of FB28 staining (Figure 5A). Of note, knockdown of *serp* or *verm* in the fat body did not cause FB28 dye diffusion in any compartment of the wing (Figure 5B).

Next, we used Eosin Y dye staining at 55 °C to investigate wing permeability in *serp* or *verm* RNAi flies. In line with previous studies [24], Eosin Y dye penetrated into two regions of the posterior wing area in control flies (Figure 5C,D). By contrast, Eosin Y penetrated into the entire posterior compartment of wings in *Gal80^ts^*, hh > *serp*-RNAi and *Gal80^ts^*, hh > *verm*-RNAi flies. However, the anterior compartment was not affected (Figure 5C). These results indicate that suppression of *serp or verm* in the posterior half of wings disrupted cuticle barrier function locally. We next asked whether suppression of *serp* or *verm* in the fat body may have any effect on wing permeability. We found that in cg > *serp*-RNAi or cg> *verm*-RNAi flies, wings exhibited the same patternof dye staining as control wings (Figure 5D). This result indicates that knockdown of *serp* or *verm* in the fat body does not affect wing permeability. Therefore, repression of these two genes in the posterior half of wings only affects the permeability of the posterior compartment but not of the anterior compartment. This suggests that Serp and Verm fulfill their function locally in the fly wing.

## 4. Discussion

### 4.1. Serp and Verm Play an Important Role in Wing Development of D. melanogaster

In general, chitin deacetylases are expressed in different chitinous tissues and exert their function in the same tissue locally. For instance, *McCDA1* is highly expressed in the intestine and the peritrophic membrane in the bertha armyworm *Mamestra configurata*. Repression of *McCDA1* caused molting deficiency in these tissues [28]. *BmCDA2* is highly expressed in silk glands and the fat body of the tobacco hornworm *Bombyx mori* and is involved in the molting process in these tissues [14]. In *D*. *melanogaster*, *serp (CDA1)* and *verm (CDA2)* have been intensively studied [18]. They function during molting, embryonic trachea formation and peritrophic membrane development [11,16,18,21,29]. The functions of *serp* and *verm* in *Drosophila* wing development have also been studied by our group [17]. We previously found during wing cuticle formation, Serp plays a major role in chitin deacetylation. However, Verm seems not needed for chitin deacetylation. In addition, Verm but not Serp is necessary for the laminar organization of chitin in the wing. In this study, we further explored the source of *serp* and *verm* in the wing.

### 4.2. Serp or Verm Function in the Wing Is Independent of Fat Body–Hemolymph Transport System

Humoral factors including hormones and metabolites are exchanged between organs during development. The insect fat body functions equivalently to the mammalian liver. Dong et al. reported that Serp was produced in the fat body and acted as a secreted factor released into the body cavity. Upon uptake by tracheal cells, Serp participates in tracheal development in the embryo [22]. In this work, we attempted to explore the source of Serp and Verm in the wing tissue. In other words, does the fat body provide these two enzymes for correct wing development? Efficient silencing of *serp* or *verm* in the fat body did not affect their expression in the wing. Consistently, suppression of *serp* and *verm* in the fat body did not cause any visible phenotype. These results indicate that Serp or Verm in the wing act independently of their expression in the fat body. The fat body–hemolymph transport system is not required for their function in the wing. During metamorphosis, the adult tissues are formed. At this stage, the fat body is decomposed and rebuilt [30]. By contrast, the embryonic fat body is fully formed during cuticle formation and tracheal differentiation. As a secreted enzyme, Serp is also expressed in epithelial cells. Why do CDAs fulfill their function locally in Drosophila wing tissue? One possibility is that accessibility of the developing wing epithelial cells to molecules is largely restricted due to the complex modeling processes occurring at their basal membrane. Simply, transcytosis may be unfunctional in wing epithelial cells.

One possibility is that wing tissue lacks destined vesicle for CDAs transport. Thus, the CDAs from hemolymph cannot be transported into the wing. Alternatively, the structure of wing is disparate from that of trachea or fat body. Wings are rich in chitin and may have more mature epithelial barriers than embryonic trachea. Therefore, the structure of wing is likely to restrict the long-range transport of CDAs and absorption of exogenous CDAs.

### 4.3. Serp and Verm Were Produced Autonomously in the Wing

We found these two enzymes are crucial for wing development in our previous work. In this study, we explored the possible transport of these two enzymes between different compartments in the wing tissue. Our results revealed that repressing these two genes using posterior specific hh-Gal4 driver only led to enhanced permeability in the posterior compartment, while, the permeability of the anterior compartment was unchanged. Hence, we deduce that *serp* and *verm* in the wing fulfill their function locally. Obviously, the developmental mechanisms involving Serp and Verm during wing development do not require assistance by the fat body. Hence, the differentiation of different tissues relies on different mechanisms of CDA distribution and localization. This study enriches the biological function of insect CDA family and advances the understanding of cuticle formation process.

## 5. Conclusions

We showed that repression of *serp* and *verm* in the fat body does not cause wing deficiency. Repressing *serp* and *verm* locallyenhanced the permeability of the respective wing cuticle but did not affect permeability in adjacent regions. Taken together, the production of Serp and Verm in the wing was autonomous and independent of the fat body. This study advances the understanding of the role of chitin deacetylase in the insect wing development.

## Data Availability

All the data generated in this work were provided in the article.
